# Evaluation of the Nasal Surgical Questionnaire for Monitoring Results of Septoplasty

**DOI:** 10.1155/2015/563639

**Published:** 2015-11-03

**Authors:** Rolf Haye, Magnus Tarangen, Olga Shiryaeva, Liv Kari Døsen

**Affiliations:** ^1^The Department of Oto-Rhino-Laryngology, Lovisenberg Diakonale Hospital, Norway; ^2^The Department of Quality, Lovisenberg Diakonale Hospital, Norway

## Abstract

Monitoring the results of surgery is important. The otorhinolaryngology department of our hospital currently uses preoperative and postoperative versions of the Nasal Surgical Questionnaire (NSQ) for continuous evaluation of nasal septoplasty. In this study, 55 patients undergoing septoplasty answered the preoperative version twice to assess the NSQ's test-retest precision, and 75 patients answered the preoperative questionnaire before and the postoperative one 6 months after surgery to evaluate the NSQ's ability to detect change in symptoms following surgery. Both the pre- and postoperative versions of the NSQ use separate visual analogue scales (VAS) to assess nasal obstruction during the day, at night, and during exercise. Other nasal symptoms are graded as secondary outcomes using 4-point Likert scales. The mean VAS scores for the two preoperative obstruction ratings were not significantly different. The scores were significantly higher than in a normal population. There were also significant differences between preoperative and postoperative ratings. The mean pre- and postoperative scores at night for those who reported complete improvement were 66.1 and 8.4, substantial improvement 74.5 and 24.2, and no improvement 83.3 and 76.4. The NSQ reliably assesses nasal symptoms in patients and may be useful for both short and long term prospective studies of septoplasty.

## 1. Introduction

The results of septoplasty are reported in many different studies, with improvement in obstruction varying from 47% to 98% [1–4]. It is, however, difficult to compare studies as so many different instruments are used to detect changes in obstruction after surgery. Few investigations are concerned with continuous monitoring of the results of nasal septoplasty. Otolaryngology departments in every hospital in Sweden report their nasal septal surgeries to a central register, which performs a six-month follow-up of the patients. The results are open to public survey [5]. As the success of surgery diminishes over time [1], the central register has increased the follow-up time to 1 year. The ear-nose-throat department of our hospital has initiated a 6-month quality control assessment using mailed questionnaires and plans to follow this up with another assessment at 4 years after surgery. Given this long follow-up period, it will be necessary to use a scoring system that does not rely on one's memory of the preoperative symptoms.

Ideally, all patients undergoing septoplasty would be recalled for a postoperative consultation, but this is neither feasible financially nor feasible in terms of human resources. The best alternative may be to implement a quality assurance program that continuously and prospectively monitors septoplasty outcomes. This can be easily done by using preoperative questionnaires and mailed postoperative questionnaires. Our intention is to recall only patients whose responses indicate no improvement or worsening of symptoms. The results will be presented on the hospital's website and individual patient results will be provided to the operating surgeon so he/she can correlate them with the surgery performed.

There are many questionnaires available; some of them only contain items related to nasal symptoms whereas others also include general quality of life items. We want to focus on the surgical results per se and would therefore prefer a questionnaire that specifically assesses nasal symptoms. The Nasal Obstruction Symptom Evaluation (NOSE) questionnaire [3] has been validated and used in many countries, but one item is difficult to translate into the Norwegian language. Questionnaires using a single visual analogue scale (VAS) for obstruction have been used in several studies [6]. We believe that use of separate and continuous scales for obstruction in different situations (day, night, and during exercise) will yield clinically relevant information about the patients' symptoms and how they change in response to surgery. Other nasal symptoms and the use of nasal medication should also be taken into account in surveying the results. We constructed the preoperative version of the Nasal Surgical Questionnaire (NSQ), which has separate VAS for obstruction during the day, at night, and during exercise, and used Likert scales for other nasal symptoms and for the use of nasal medication [7]. The preoperative questionnaire was favourably assessed in normal volunteers but has not yet been evaluated in a patient population. For the purpose of this study, a postoperative version of the NSQ (NSQ after operation) (see the questionnaire) has also been developed. It is the purpose of this study to assess both versions of the NSQ in septoplasty patients in order to evaluate the precision of the instrument and its ability to detect change in pre- and postoperative symptoms.

## 2. Materials and Methods

The study was conducted at Lovisenberg Diakonale Hospital in Oslo, Norway, and was approved by the ethical committee of the hospital. Included in the study are patients 16 years of age or older undergoing septoplasty with or without surgery to the inferior concha. The exclusion criteria were inadequate command of the Norwegian language, any other concomitant nasal or sinus surgery, and any other nasal disease except nasal allergy.

The pre- and postoperative versions of the NSQ have separate VAS for obstruction during day, night, and exercise. The scales are 10 cm long with markings of* 0 = completely open* and* 10 = completely obstructed* at either end. The patients are asked to mark their sense of obstruction on this scale. Scores are the distance between the mark and the left end of the line (measured in mm) and can range from 0 to 100. The VAS scores are measured and recorded manually, whereas answers to the rest of the questions, which are marked in boxes, are recorded automatically by scanning. There are 4-point (1 = no, 2 = mild, 3 = moderate, and 4 = severe) Likert scales for five other nasal symptoms (crusting, bleeding, sneezing, secretion, and nasal pain) and for nasal medication (vasoactive drugs, topical steroids, and antihistamines). In addition, the instrument includes items about smoking habits and allergy. The postoperative NSQ is supplemented with the following 5-point retrospective rating of perceived improvement: Is your nasal breathing completely, substantially, mildly, or not improved, or has it deteriorated? Patients are asked to answer the questionnaire based on a normal day without nasal infection. It was intended to be self-explanatory and to a large extent it was. However, in some cases, we had to instruct the patients in how to complete the questionnaire and in a few others we had to call up the patients to clarify ambiguous responses. As a result of this, we began checking each questionnaire preoperatively.

To ensure the quality of the manual VAS scoring, the markings of the scales of 50 patients were rescored by a second person. The mean difference in scores between the first and the second measurements was 0.9 (0–5) on a scale from 0 to 100. In only two cases was the difference in scores more than 2 mm. Ten percent of the patients recorded the scores on the scale with numbers with or without a mark. For these, we recorded the number and not the marking.

### 2.1. Test-Retest Precision

During 2014, the preoperative version of the NSQ was administered twice to patients who were scheduled for septoplasty with or without surgery to the inferior concha. They were administered at least four weeks apart to ensure that patients would not recollect the precise location of their responses to the first questionnaire. At the time of the second preoperative questionnaire, the patients were also asked if they regarded the intensity of their nasal symptoms as stronger, weaker, or equal to the first questionnaire. Only those who perceived their symptoms to be equal on both occasions were included in the test-retest analysis. Patients who had received treatment for nasal symptoms in the interim period were also excluded. Both positive and negative differences between the two questionnaires were given a positive numerical value. To reduce the influence of nasal allergy on the results, we collected most of the questionnaires for this study from October 2013 to April 2014 and from October to December 2014.

### 2.2. Results of Surgery

All patients who had adequately answered the questionnaire preoperatively were presented with the postoperative questionnaire by mail six to eight months after surgery. Only patients who had their septoplasty performed endonasally were included in the analysis. Included patients were operated on from February to mid-August 2014, and the postoperative questionnaires were collected in the pollen-free time, October 2014 to February 2015, 6–8 months after surgery. Patients in our department are never operated on during their pollen allergy season.

### 2.3. Statistical Analysis

Continuous data are presented as means and standard deviation (SD) and categorical data as frequencies and percentages. Group comparisons of VAS scores were performed using the Mann-Whitney *U* test. The Wilcoxon Signed Ranks Test was applied to evaluate the difference in responses between the first and second presentation of the preoperative version of the questionnaire and between the pre- and postoperative versions on the VAS items for nasal obstruction during the day, at night, and during exercise. For the other nasal symptoms measured on a four-point scale, marginal homogeneity tests were performed to estimate the difference in responses between the first and second questionnaire. Cronbach's alpha was used to assess the internal consistency of the three VAS items (at day, at night, and during exercise). Data were analyzed with SPSS software (version 22.0 for Windows, IBM Corp., Armonk, NY). All tests were two-sided, and *p* values < 0.05 were considered statistically significant.

## 3. Results

### 3.1. Test-Retest Precision

Fifty-five patients, 37 male and 18 female with a mean age of 40.4 (SD 12.9) years, completed the preoperative version of the NSQ twice prior to surgery. There were 8 smokers and 18 with self-reported nasal allergy. The results from both preoperative questionnaires are presented in Table 1. The sample sizes vary because a few patients left one or more questions unanswered. The three different VAS items were highly correlated, both in the first (Cronbach's alpha = 0.824) and in the second (Cronbach's alpha = 0.800) questionnaire. For both questionnaires, the mean score for obstruction at night was significantly higher than during the day (*p* < 0.001) and during exercise (*p* < 0.05). The mean difference in scores between the first and second questionnaire was 7.7 (SD 5.7) for day, 6.9 (SD 5.8) for night, and 8.5 (SD 7.7) for exercise. The Wilcoxon Signed Ranks Tests showed that there was no significant difference between these scores. The Mann-Whitney *U* test showed that the scores for males versus females, smokers versus nonsmokers, and allergic patients versus nonallergic patients were not significantly different. The Likert scale scores for the other nasal symptoms and nasal medication were not significantly different between the first and second questionnaire.

### 3.2. Results of Surgery

Of the 102 patients who had responded to the preoperative questionnaire and were operated for nasal septal deviation with or without surgery to the inferior concha, 75 (73.5%) answered the postoperative questionnaire and were included in the comparison of pre- and postoperative scores. These 75 patients included 48 males and 27 females and had a mean age of 41.6 (SD 13.8) years. There were 14 (19%) smokers and 23 (33.8%) with self-reported allergy.

The mean preoperative and postoperative VAS obstruction scores indicated significant improvement following surgery: 62.8 versus 33.8 for day, 75.6 versus 37.9 for night, and 65.0 versus 37.9 for exercise, respectively, all *p* < 0.05. The three VAS scales were correlated with each other both preoperatively (Cronbach alpha = 0.79) and postoperatively (0.96). We compared the VAS scores with the 5-point retrospective rating of improvement (complete, substantial, mild, or no improvement or worsening of symptoms). The results for preoperative, postoperative, and improvement scores are presented in Table 2. The mean differences in VAS scores before and after the operation compare favourably to the retrospective rating of improvement. There was no statistically significant difference in VAS scores for day, night, or exercise between males and females, smokers and nonsmokers, and allergic and nonallergic patients, in either the preoperative or postoperative questionnaire.

The pre- and postoperative differences in the 4-point Likert scale scores for the other nasal symptoms are shown in Table 3. There was a statistically significant change in scores for bleeding, crusting, sneezing, and nasal pain (*p* < 0.05). Most of the differences indicate symptom improvement but a few patients had worse symptoms postoperatively.

## 4. Discussion

The results of these two analyses provide evidence of the NSQ's precision in assessing nasal symptoms and its ability to detect change in symptoms following nasal surgery. These findings suggest that it may be a useful tool for efficiently monitoring surgery results and identifying patients who need further follow-up.

The VAS obstruction scores obtained in this study using the NSQ are comparable to those reported in other studies. Rhee and colleagues [6] have established normative VAS obstruction scores in normal people and patients before and after nasal surgery by critically reviewing prior studies using VAS to assess nasal obstruction. One of the studies [8] used four-point scales for nasal obstruction, which were converted to VAS scores of 0 to 10. To facilitate comparison with our results, we have taken the liberty of further converting the normative VAS scores to a scale from 0 to 100. All of the reviewed studies used only a single VAS item to assess nasal obstruction and did not distinguish between symptoms experienced during the day, at night, or during exercise. The mean preoperative VAS score in these articles [4, 8–15] was 67, which is comparable to the VAS scores we obtained using the NSQ. In our studies using three items to assess obstruction in different situations, the preoperative VAS score for obstruction at night was significantly higher than the scores for day and exercise. In the present test-retest study, these scores from the first questionnaire were 73.7 for night, 61.8 for day, and 66.2 for exercise. Given the relatively high scores, symptoms during the night may be the most important for patients and should be included in future studies. Our study has shown that the differences in nasal obstruction scores between first and second administration of the NSQ were very small, only 7.7 for day scores, 6.9 for night, and 8.5 for exercise. We have not found comparable data in the other articles [4, 8–15].

Our evaluation of the results of surgery showed that the patient's short term (6 months) retrospective perception of their improvement in breathing was comparable to the prospective VAS scores. Not surprisingly, patients who reported their breathing as completely improved had mean VAS score close to 0 postoperatively. The same is apparent in those who rated their breathing as substantially improved; their mean VAS scores were 20.9 (day) and 24.2 (night), regardless of the relative improvement in scores. Patients reporting mild improvement scored 65.5 (day) and 76.7 (night) preoperatively and improved by 21.2 and 27.2, respectively. These results lend credence to the values for both the 6-month retrospective improvement rating and the prospective change in VAS scoring. For short term results being presented to the public, it may be sufficient to use a 5-point retrospective scale. For our prospective long term studies, however, we believe that recollection of the state of their preoperative obstruction will be spurious. We found that the prospective VAS scoring reliably reflects the patients' assessment of their obstruction. At 3 to 5 years this prospective recording will be unaffected by patients' recollection.

Although there were differences between the VAS scores for obstruction during the day, at night, and during exercise, the scores were highly correlated. One or two of the scales may therefore be superfluous. As the highest scores are seen at night, one might choose this as the scale best representing the patients' situation. There are, however, patients who are more bothered with symptoms during the day or during exercise than at night. The patient reporting worse symptoms after surgery, for instance, had a negative change in the VAS score during the day and no change at night. Individual differences may not influence the overall group score but may be relevant to the surgeons. We will therefore continue with separate scales.

Rhee et al. [6] did not identify the minimal clinically significant difference in VAS score that would signify an improvement after therapy. However, we have found that the change in VAS scores after surgery in the patients reporting mild improvement was 21.2 during the day and 27.2 at night. Patients reporting no improvement had a mean difference in score of 2.7 during the day and 7.3 at night. In a prior study [7] using the NSQ in a normal population, we found that the mean change in scores between two administrations was 5.09 during the day and 6.22 at night. The present study showed that the score between the two preoperative administrations were 7.7 for day and 6.9 at night. Together, these data indicate that the minimal significant change in VAS score is likely somewhere between 10 and 20 during the day and at night. We are not aware of other attempts to identify this, and further studies are needed to supplement these results.

Questionnaires, such as the NSQ, are often used for group comparisons, but differences across studies can make comparisons between them difficult. In prior studies [4, 8–15], patients typically served as their own control, but personal factors, such as gender, age, weight, smoking, and allergy, may still influence the results [16], particularly if study populations vary considerably in composition. The individual result of surgery may also depend on the influence of these other factors. Studies have also included different surgical procedures, such as rhinoplasty, septoplasty, turbinoplasty, polypectomy, and sinus surgery, used either alone or in combination, which may have influenced the results reported. Two studies [8, 14] only included patients with allergy, another [12] had patients rate their preoperative symptoms retrospectively, and a fourth [13] showed patients their preoperative ratings before asking them to rate their postoperative symptoms. In our study, we asked the patients to answer the questionnaire based on a normal day when they were free of nasal infection to eliminate spontaneous variations and the influence of nasal infection. However, it is not clear whether other studies used a similar approach. These differences need to be considered when comparing findings with the current study.

Smoking may reduce surgery results, as clinical studies have shown that smoking negatively influences nasal breathing [17–19]. This, however, was not apparent in our study, probably because the structural deformities were so prominent preoperatively and still present to various degrees postoperatively. To overcome the influence of smoking, one study excluded smokers [14]. Other studies have not examined the influence of smoking.

A large part of the population has allergy, which may influence the results of surgery [20]. Two studies addressed this problem by only including patients with allergy [8, 14], while other studies took no measures to circumvent the confounding influence of allergy [4, 9–13, 15]. For this study, we have taken care to reduce the influence of allergy by collecting the questionnaire outside of the pollen seasons. However, as we plan to run permanent quality control throughout the year, this problem may occur and we have therefore included questions about allergy status and the use of medication in the NSQ. This will be helpful in assessing the results, which may vary by time of year. To reduce the influence of allergy, it might be preferable to postpone the postoperative assessment from 6 to 12 months after the surgery so that influence of allergy season will be the same for both the pre- and postoperative assessments.

A study using only allergic patients compared the result of septoplasty with or without turbinoplasty [8]. There was no change in scores, regardless of whether the inferior concha had been operated upon, but the use of nasal steroid medication did substantially decrease after surgery. They did not evaluate the quantitative influence that the medication might have had on nasal obstruction. A patient who is able to stop taking medication may be satisfied, even though the obstruction is not completely relieved. The use of nasal medication was also reduced in our study.

Our surgeons would like to review not only the overall results of surgery but also the individual pre- and postoperative questionnaires for assessing their surgical praxis. The NSQ allows them to take into account not only the VAS scores but also the change in the intensity of the other nasal symptoms and the use of medication.

In a prior study [7], we evaluated the preoperative NSQ in a normal population. Many persons had no breathing problem and scored 0 on the VAS, whereas others had some nasal obstruction for which they did not seek medical attention. The mean VAS scores for obstruction in the normal population were 9.99 during day, 12.95 at night, and 11.67 during exercise. When we compare these to the preoperative scores in surgical patients, we find that the preoperative NSQ significantly differentiates between patients and normal persons. This is reconfirmed by the substantial change in scores after surgery.

## 5. Conclusion

We developed the pre- and postoperative NSQ to prospectively and continuously monitor the results of septoplasty. The difference in VAS scores for nasal obstruction between two preoperative administrations of the NSQ was minimal, indicating reliability and precision in scoring. The changes in VAS scores after surgery were comparable to the retrospective improvement ratings, indicating that the VAS scoring is representative of the results. There is also a substantial difference between preoperative scores in patients and those seen in a normal population, indicating that the NSQ has useful discriminatory power. These findings indicate that the NSQ may be useful for short and long term quality control of nasal septal surgery. 

*Nasal Surgical Questionnaire after Operation (Answer When Free of a Cold/Nasal Infection)*

*Is Your Nasal Breathing*

□ Completely improved
□ Substantially improved
□ Mildly improved
□ Unchanged
□ Worse

**Table:** 

Rate your sense of obstruction	Open	Put a mark on this scale (*0* =* completely open. 10* =* completely blocked*.)	Blocked
On a normal day	0	–––––––––––––––	10
At night	0	–––––––––––––––	10
During exercise	0	–––––––––––––––	10

*Rate These Nasal Symptoms*

*Crusting*

□ None
□ Slight
□ Moderate
□ Severe

*Bleeding*

□ None
□ Slight
□ Moderate
□ Severe

*Sneezing*

□ None
□ Slight
□ Moderate
□ Severe

*Secretion *

□ None
□ Slight
□ Moderate
□ Severe

*Nasal Pain*

□ None
□ Slight
□ Moderate
□ Severe

*Rate Your Use of Nasal Medication*

*Nonprescriptional Nasal Spray/Drops (Naso/Nazaren/Otrivin/Rhinox/Zymelin/Zycomb)*

□ None
□ Slight
□ Moderate
□ Daily

*Corticosteroid Nasal Spray/Drops (Avamys/Budesonid/Flutide nasal/Nasacort/Nasonex/Rhinocort)*

□ None
□ Slight
□ Moderate
□ Daily

*Antihistamines (Aerius/Alzyr/Cetrizin/Clarityn/Kestine/Loratadin/Telfast/Zyrtec/Xyzal)*

□ None
□ Slight
□ Moderate
□ Daily

*Smoking*

□ None
□ 1–10 daily
□ 11 or more daily

*Do You Suffer from Nasal Allergy*

□ Yes
□ No
□ Uncertain

 If yes
 do you have nasal allergy at present
  □ Yes
  □ No
 do you use allergy medication at present
  □ Yes
  □ No

## Figures and Tables

**Table 1:** Preoperative test-retest VAS scores.

	Questionnaire 1			Questionnaire 2		
	*N*	VAS (mean)	[SD]	*N*	VAS (mean)	[SD]
Day	55	(61.8)	[20.4]	55	(63.4)	[18.8]
Night	55	(73.7)	[18.3]	54	(74.3)	[16.8]
Exercise	52	(66.2)	[24.4]	53	(65.7)	[24.2]

**Table 2:** Results of surgery. Pre- to postoperative change in day and night VAS scores for each level of perceived improvement in nasal breathing.

Perceived improvement	*N*	Day VAS scores			*N*	Night VAS scores		
		Preop	Postop	Difference		Preop	Postop	Difference
		Mean (SD)	Mean (SD)	Mean (SD)		Mean (SD)	Mean (SD)	Mean (SD)
Complete	8	57.8 (19.3)	5.9 (5.1)	51.9 (21.0)	8	66.1 (21.6)	8.4 (6.2)	57.8 (22.1)
Substantial	34	60.9 (19.7)	20.9 (16.5)	39.9 (24.4)	34	74.5 (19.4)	24.2 (15.9)	50.3 (23.3)
Mild	21	65.5 (17.4)	44.3 (15.8)	21.2 (16.2)	19	76.7 (15.0)	49.9 (18.6)	27.2 (17.1)
No	11	69.5 (22.3)	72.3 (19.8)	2.7 (15.2)	10	83.3 (16.7)	76.4 (25.9)	7.3 (13.6)
Worse	1	33.0 (—)	47.0 (—)	14.0 (—)	1	88.0 (—)	87.0 (—)	1.0 (—)
Total	75	62.8 (19.6)	33.8 (25.7)	29.0 (27.0)	72	75.6 (18.2)	37.9 (27.9)	38.4 (26.4)

**Table 3:** Differences between pre- and postoperative scores on the 4-point Likert scales for other nasal symptoms.

Symptoms	*N*	Deterioration		No change	Improvement		
		2 points	1 point	0 points	1 point	2 points	3 points
		*N* (%)	*N* (%)	*N* (%)	*N* (%)	*N* (%)	*N* (%)
Crusting	72	1 (1.4)	10 (13.9)	36 (50.0)	16 (22.2)	6 (8.3)	3 (4.2)
Bleeding	74	2 (2.7)	7 (9.5)	38 (51.4)	19 (25.7)	6 (8.1)	2 (2.7)
Sneezing	69	1 (1.4)	7 (10.1)	32 (46.4)	20 (29.0)	9 (13.0)	0 (0.0)
Secretion	73	2 (2.7)	15 (20.5)	31 (42.5)	16 (21.9)	8 (11.0)	1 (1.4)
Nasal pain	72	1 (1.4)	6 (8.3)	41 (54.7)	20 (27.8)	3 (4.2)	1 (1.4)

Statistically significant changes for all symptoms (*p* < 0.05, marginal homogeneity tests) except for secretion.
